# Novel functions of the luteinizing hormone/chorionic gonadotropin receptor in prostate cancer cells and patients

**DOI:** 10.1371/journal.pone.0238814

**Published:** 2020-09-03

**Authors:** Hein Vincent Stroomberg, Anne Jørgensen, Klaus Brasso, John Erik Nielsen, Anders Juul, Hanne Frederiksen, Martin Blomberg Jensen, Martin Andreas Røder

**Affiliations:** 1 Copenhagen Prostate Cancer Center, Department of Urology, Rigshospitalet, Copenhagen University Hospital, Copenhagen, Denmark; 2 Group of Skeletal, Mineral and Gonadal Endocrinology, Department of Growth and Reproduction, Rigshospitalet, Copenhagen, Denmark; 3 Department of Growth and Reproduction and International Center for Research and Research Training in Endocrine Disruption of Male Reproduction and Child Health (EDMaRC), Rigshospitalet, University of Copenhagen, Copenhagen, Denmark; 4 Division of Bone and Mineral Research, HSDM/HMS, Harvard Medical School, Boston, MA, United States of America; Universita degli Studi della Campania Luigi Vanvitelli, ITALY

## Abstract

Prostate cancer (PCa) cells become castrate-resistant after initial tumor regression following castration-based lowering of testosterone (T). De-novo intra-tumoral steroid synthesis is a suggested biological mechanism of castration resistant PCa, but the regulators are unknown. Testicular T production is controlled by the luteinizing hormone/choriogonadotropin receptor (LHCGR). To elucidate the influence of LHCGR on PCa development the presence and effects of LHCGR in PCa and whether LHCGR in serum holds prognostic information in PCa patients is investigated. LHCGR expression was investigated by RT-PCR, WB, IHC, qPCR in PCa cell lines and prostatic tissue. Steroid production was measured in media from cell lines with LC-MS/MS and expression of steroidogenic enzymes with qPCR. Serum LHCGR (sLHCGR) was measured with ELISA in PCa patients (N = 157). Presence of LHCGR was established in prostatic tissue and PCa cell lines. Cell proliferation increased by 1.29-fold in LNCaP (P = 0.007) and 1.33-fold in PC-3 cells (P = 0.0007), when stimulated by luteinizing hormone. Choriogonadotropin stimulation decreased proliferation 0.93-fold in DU145 cells (P = 0.05), but none of the treatments altered steroid metabolite secretion. Low sLHCGR concentration was associated with a higher risk of biochemical failure after radical prostatectomy (HR = 3.05, P = 0.06) and castration resistance (HR = 6.92, P = 0.004) compared to high sLHCGR concentration. LHCGR is expressed in PCa and may exert a growth regulatory role in PCa derived cell lines. A potential prognostic role of sLHCGR for determining recurrence risk in PCa patients is found in this pilot study but needs verification in larger cohorts.

## Introduction

Prostate cancer (PCa) cells are sensitive to testosterone (T) and the removal of T by medical or surgical castration results in tumor regression [1, 2]. However, within a median of 2–3 years PCa cells can become castration resistant (CRPC) in a process driven by multiple pathways, but critically dependent on sex steroid signaling. Several aberrations have been suggested, including upregulation or mutations of the androgen receptor (AR) and initiation of intratumoral steroid synthesis [2]. Novel treatment options for CRPC such as Enzalutamide, an AR antagonist, and Abiraterone acetate, a CYP17A1 antagonist, have emerged, but does not exert long-term disease control. Consequently, there is a need to understand the biological processes underlining CRPC to develop new treatment strategies and for biomarkers that refine prediction of which patients will benefit from current or coming treatment options [3, 4].

In males T production is controlled by luteinizing hormone (LH) that binds to the luteinizing hormone/choriogonadotropin receptor (LHCGR) in the Leydig cells. T released to serum is subsequently converted to dihydrotestosterone in the prostate [5, 6]. Men and rodent models with no LH production or inactivating mutations in LHCGR have very low circulating T and as a result a very low prostate volume [7]. The gene encoding the G-protein coupled receptor (GPCR) LHCGR is located on chromosome 2, consists of 11 exons with exon 1–10 encoding extracellular and exon 11 for transmembrane and intracellular carboxy-terminal tail domains [5]. LHCGR has two known endogenous agonists in men, LH during adult life and human choriogonadotropin (hCG) during fetal development, which upon binding stimulate steroidogenesis. Several isoforms of LHCGR have been identified both at the transcriptional and protein level in the testes and some extracellular tissues [8]. The LHCGR isoform comprising the whole extracellular domain including the LH/hCG binding sites but lacking the transmembrane and intracellular domains may be secreted to the blood stream [8–11]. Analyses of serum and seminal fluid from young men showed that the size of LHCGR isoforms were similar to the size of isoforms found in the testes. Indicating that, if not the full receptor, then a large protein isoform with most or all the extracellular domain of the LHCGR is excreted and may bind LH/hCG in serum [8, 11]. A recent study suggested that serum LHCGR levels may have prognostic value in seminoma patients [12]. At our hospital, we identified a patient without testicles but with very high circulating LHCGR that had developed PCa which led us to speculate that LHCGR could be expressed and potentially released from PCa cells. Additionally, a polymorphism in LHCGR that increase its activity was previously associated with decreased survival in men, a few reports have found LHCGR expression and LH-activity in PCa derived cell lines, and expression of hCG in prostate tumors has been associated with poor prognosis, which all supports a role for LHCGR in PCa progression [13–16]. This led us to investigate expression of LHCGR in PCa derived cell lines, PCa tissue from patients, and test the effect of LH and hCG on tumor growth and steroidogenesis. Further, we measured LHCGR in serum from PCa patients to determine its potential as biomarker for disease progression.

## Methods

### Cell lines

The DU145 (RRID:CVCL_0105), PC-3 (RRID:CVCL_0035) and LNCaP (RRID:CVCL_0395) cell lines were gifts from Professor Lene Juel Rasmussen (Center for Healthy Ageing, University of Copenhagen), the cells were authenticated with the human cell line authentication service from Eurofins Genomics at 17-12-2019. From thawing, a minimum of 3 passages and a maximum of 10 passages were performed before start of an experiment and passage number used in experiments ranged from 9 to 40. Mycoplasma tests (#LT07-118, MycoAlert™, Lonza, Basel, Switzerland) were performed every 4–6 weeks according to manufactures instructions. The LNCaP and PC-3 cell lines were grown in Roswell Park Memorial Institute (RPMI 1640, Gibco™, Waltham, USA) supplemented with 10% fetal bovine serum (FBS, Gibco™), 2 mM L-Glutamine (Gibco™) and 1% Penicillin/Streptomycin (P/S, Gibco™). The DU145 cell line was grown in Dulbecco’s modified Eagle medium (DMEM, Gibco™) supplemented with 10% FBS (Gibco™), 2 mM L-Glutamine (Gibco™) and 1% P/S (Gibco™). All cell lines were incubated at 37°C with a CO_2_ concentration of 5%. Cells were harvested by dissociating the cells with 1x Trypsin (Gibco™) diluted from a 10x solution in Phosphate-buffered Saline (PBS, Gibco™). Hereafter the cells were either counted using NucleoView NC-3000™ (Chemometec A/S, Lillerød, Denmark) with VIA-1 cassettes™ (Chemometec A/S) and reallocated into wells for a proliferation assay or stored at -80°C for protein and RNA purification.

### Western blot

Protein purification and Western blotting (WB) was performed as described previously [17]. In brief, protein was purified from snap-frozen samples and cell pellets by homogenization in a lysis buffer supplemented with protease (Roche, Basel, Switzerland) and phosphatase inhibitors (Thermofisher scientific, Waltham, USA). 15 μg of protein was loaded in each lane. Primary antibodies were: LHCGR (c-terminal targeting, Aviva, # OASG04237), LHCGR (n-terminal targeting, Promab # Pro-30751, Ramlösa, Sweden) and β-actin (loading control, Santa Cruz, # sc-47778, Dallas, USA) all in a concentration of 1:200. Anti-rabbit (Dako, # P0217, Glostrup, Denmark), anti-goat (Dako, # P0160) and anti-mouse (Dako, # P0260) secondary antibodies were horseradish peroxidase (HRP) conjugated and used in a 1:500 dilution followed by development using ultra-sensitive enhanced chemiluminescent (Thermofisher scientific). Pictures were taken using ChemiDoc™ MP imaging system (Bio-rad, Hercules, USA) with automatic exposing time for intense bands. Experiments were repeated at least three times in independent experiments.

### Reverse Transcription-/quantitative Polymerase Chain Reaction

RNA was purified using the NucleoSpin® RNA isolation kit from Macherey Nagel™ (Düren, Germany) according to manufacturer’s protocol and concentrations of RNA were measured using a Nanodrop (Thermofisher scientific). Using 1 μg of mRNA together with dt20 primer and random hexamers, cDNA was synthesized as previously described [18].

Reverse Transcription-Polymerase Chain Reaction (RT-PCR) was conducted on GeneAmp® PCR system as previously reported [19]. Specific primers targeting genes of interest (Table 1) were designed to span intron-exon boundaries and all amplicons were verified by sequencing (Eurofins genomics, Ebersberg, Germany). For RT-PCR 1 to 5 μl of cDNA were used and testis tissue samples served as a positive control, while sterile H_2_O was included as a negative control. The PCR conditions were: 95°C for 3 min followed by 40 cycles of 30 seconds at 95°C, 1 minute at a specific annealing temperature and 1 minute at 72°C. The annealing temperature depended on the primers (see details in Table 1). Pictures were taken using the ChemiDoc™ MP imaging system (Bio-rad).

**Table 1:** Overview of primers used for RT-PCR and qPCR. Length represents the expected band size and this band has been validated by sequencing.

Primer Target		Sequence (5’-3’)	Length	Annealing temp.	Melt curve temp.
*hLHCGR* exon 1 to 4	Forward	CCTACCTCCCTGTCAAAGTG	205 bp	60°C	
	Reverse	ATGCTCCGGGCTCAATGTATC			
*hLHCGR* exon 11	Forward	CGATTTCACCTGCATGGCAC	360 bp	60°C	60°C
	Reverse	GTGTAGCGAGTCTTGTCTAG			
*hCYP11A1*	Forward	CTGCATCTTCAGTCGTCTGTC	83 bp	58°C	65°C
	Reverse	GGTGACCACTGAGAACCCATTC			
*hSTaR*	Forward	CACCCCTAGCACGTGGATTA	152 bp	60°C	
	Reverse	CTTGGTTGCTAAGGATGCCC			
*hCYP17A1*	Forward	TCCCCAAGGTGGTCTTTCTGAT	110 bp	58°C	65°C
	Reverse	GTGGACAGGGGCTGTGAGTTAC			
*hHSD3B1,2*	Forward	GGGCCCAACTCCTACAAGGA	356 bp	58°C	
	Reverse	ACTTGGGGCCTTCTTGGGGT			
*hAKR1C3*	Forward	GGAGGCCATGGAGAAGTGTAAGGA	215 bp	62°C	
	Reverse	CCAGAGCACTATAGGCAACCAGAAC			
*hCYP21A2*	Forward	GAGTTCTGTGAGCGCATGAG	205 bp	60°C	
	Reverse	GAATCACGTCCACAATTTGGAT			
*hCYP11B1,2*	Forward	CTTCCACTACACCATAGAAGCCAGC	200 bp	60°C	
	Reverse	CCTCAAAGTGCTCCTTCCACAC			
*hCYP19A1*	Forward	GTCACTCTTGAATGTGCAATGT	192 bp	55°C	60°C
	Reverse	GGGAAAATGGGATCTCAATGAA			
*hRPS20* (RT-PCR)	Forward	AGACTTTGAGAATCACTACAAGA	179 bp	62°C	
	Reverse	ATCTGCAATGGTGACTTCCAC			
*hRPS20* (qPCR)	Forward	AACAAGCCGCAACGTAAAATC	166 bp	62°C	60–65°C
	Reverse	ACGATCCCACGTCTTAGAACC			

Quantitative RT-PCR (qPCR) was run using the QuantStudio 3 Real-Time PCR System (Thermofisher scientific) as previously described [19]. In short, the qPCR analysis was set up in biological and technical triplicates using the Brilliant II SYBRR GREEN QPCR Master Mix (Agilent, Santa Clara, USA) and 1 or 4 μl cDNA per sample. The PCR conditions were: a hold stage at 95°C for 15 minutes followed by a PCR stage of 40 cycles of 15 seconds at 95°C and 1 minute at a specific annealing temperature, followed by melt curve stage of 15 seconds at 95°C, 1 minute at 60–65°C and 1 second at 95°C. The annealing temperatures and a melt curve stage temperature depended both on the primer used (Table 1). From this data relative gene expression was calculated using the *RPS20* gene as a reference. The relative gene expression was calculated using the ΔΔCt method and shown as a ratio relative to the appropriate control group which was set to 1 [20].

### Immunohistochemistry (IHC)

For IHC deparaffinized formalin fixed and paraffin embedded PCa tissue and fixed PCa cell lines grown on Lab-TECII chamber slides (154526, Nalge Nunc international, Naperville IL 60563–1796, USA) were used. Duplicate PCa tissue slides were obtained from the pathology department of patients with confirmed PCa as defined by a trained pathologist. The protocol used for IHC was previously described in [21]. It only diverted for the deparaffination step. In short, to damask the cells and tissue they were heated with TEG buffer (Tris 6,06g, EGTA 0,959 in 5L, pH 8,5) in a pressure cocker (Biocare medical decloaking chamber, Concord, Ca, USA). The slides were then blocked with 1% (v/v) H_2_O_2_ in MeOH for 30 minutes and with 5% BSA (w/v) in horse serum (20% v/v) (ImmPRESS, Vector Laboratories, Burlingame, CA) and Tris buffered saline (80% v/v) before application of antibody. The LHCGR OASG04237 antibody (ProMab biotechnologies Inc. Catalog # 30751) was diluted 1:7500 for the tissue and 1:200 for the cell lines and left overnight at 4°C followed by 1 h at room temperature. Anti-mouse secondary antibody (ImmPRESS, MP-7402) was applied for 30 minutes at room temperature and development was achieved with AEC (3-amino-9-cabazole, ImmPRESS, SK4205). Omission of primary antibody was used as control on sections of prostate, testis tissue and the cell lines.

### Cell stimulation and proliferation assay

LNCaP, PC-3 and DU145 cells were plated in 6-well plates at 0.5*10^6^ cells per well for stimulation and 96-well plates with 7000 cells per well for proliferation. Cells were stimulated with recombinant LH (1636 pM = 1 IU/ml, Luveris, Merck Serono, Rome, Italy) or urinary purified hCG (1947 pM = 1 IU/ml, Prospecbio, # HOR-250, Rehovot, Israel) diluted into the normal cell culture media DMEM for DU145 and RPMI for LNCaP and PC-3, concentrations were chosen based on previous research [14]. In each proliferation experiment 12 wells without cells were included to serve as blank samples. The cells were grown for 48 hours and harvested for further analysis or used in a cell proliferation assay (BrdU, Roche, # 11647229001) according to the manufacturer’s instructions.

### Steroid analysis

Androgens and corticosteroids were analyzed in media from LNCaP cells (RPMI 1640) and DU145 cells (DMEM) using isotope diluted TurboFlow-liquid chromatography-tandem mass spectrometry (LC-MS/MS) for quantitation of progesterone, corticosterone, 17-hydroxy progesterone (17-OHP), 11-deoxycortisol, cortisol, cortisone, dehydroepiandrosterone sulfate (DHEAS), androstenedione, T and estrone sulphate (E1-S) according to a method previously described [22] with minor modifications for measurements of androgens and corticosteroids in cell media. Limit of quantification (nmol/L) based on validation in adult cell media (DMEM) were: progesterone, 0.04 nmol/L; corticosterone, 0.28 nmol/L; 17-OHP, 0.08 nmol/L; 11-deoxycortisol, 0.04 nmol/L; cortisol, 0.58 nmol/L; cortisone, 0.16 nmol/L; DHEAS, 1.02 nmol/L; androstenedione, 0.14 nmol/L; T, 0.08 nmol/L; E1-S, 0.08 nmol/L. The relative standard deviation was determined in cell culture media spiked with a mixture of low and high concentrations of androgens and corticosteroids and ranged from 0.11–6.9% and 1.2–6.4% for all analytes. The spiked cell culture media (RPMI 1640 and DMEM) was supplemented with FBS and P/S.

### Patient information

The research on patient samples has been approved by the Danish National Committee on Biomedical Research Ethics for the Capital Region (Tissue no.: H-6-2014-111; Serum no.: H-4-2011-071). Blood samples were collected by vein puncture at the time of diagnosis after written informed consent. Sampling was done at the laboratory at Rigshospitalet, Denmark. Blood was collected in a citrate and a Na2-EDTA tube. Separation of plasma from blood were done within 30 min after vein puncture by centrifugation at 3000*rpm at 4°C for 10 min. After the centrifugation, the samples were immediately stored in aliquots of 200 μl in cryotubes at -80°C until analyzed. All serum samples were part of the CuPCa database that previously has been described in detail [23].

### ELISA

To measure total serum LHCGR (both in complex with LH/hCG and not in complex), an ELISA (Novel Biomarkers Catalyst Lab (NBCL), Holland, detection limits: from 0.01 to 15.55 pmol/L) was performed using a previously validated and described method [24]. In brief, a 96-well plate was coated with purified Aviva LHR-29 antibody that was also used for the WB analysis. Plates were blocked at room temperature with phosphate buffered saline containing 1% casein concentrate. Serum from patients was added followed by washing of the plates. Next, secondary horseradish peroxidase-conjugated LHR29 antibodies were added followed by detection using TMB substrate (Pierce, # 34021) and read out by 450–620 nm.

### Statistics

Differences in proliferation and gene expression levels were tested for normality both visually and with the Shapiro-Wilk test, followed by analysis of variance (ANOVA) and a post hoc analysis of student t-tests or Kruskal Wallis test with post hoc Wilcoxon signed rank test without adjustment for multiple comparisons. The cumulative incidence curves for the patient data are generated with the “survival” package in R, univariate and multivariate cox regressions were performed for hazard of biochemical failure (BF) after radical prostatectomy (RP) and of castration resistance after castration, results are presented as hazard ratio’s (HR) and 95% confidence intervals (CI). In the multivariate analysis the outcome was adjusted for age and PSA at diagnosis, Gleason score and tumor size. Significance was defined as a P-value of 0.05 or below. All statistical analyses were done using the statistical program R.

## Results

### Presence of LHCGR in PCa and derived cell lines

**Fig 1:**
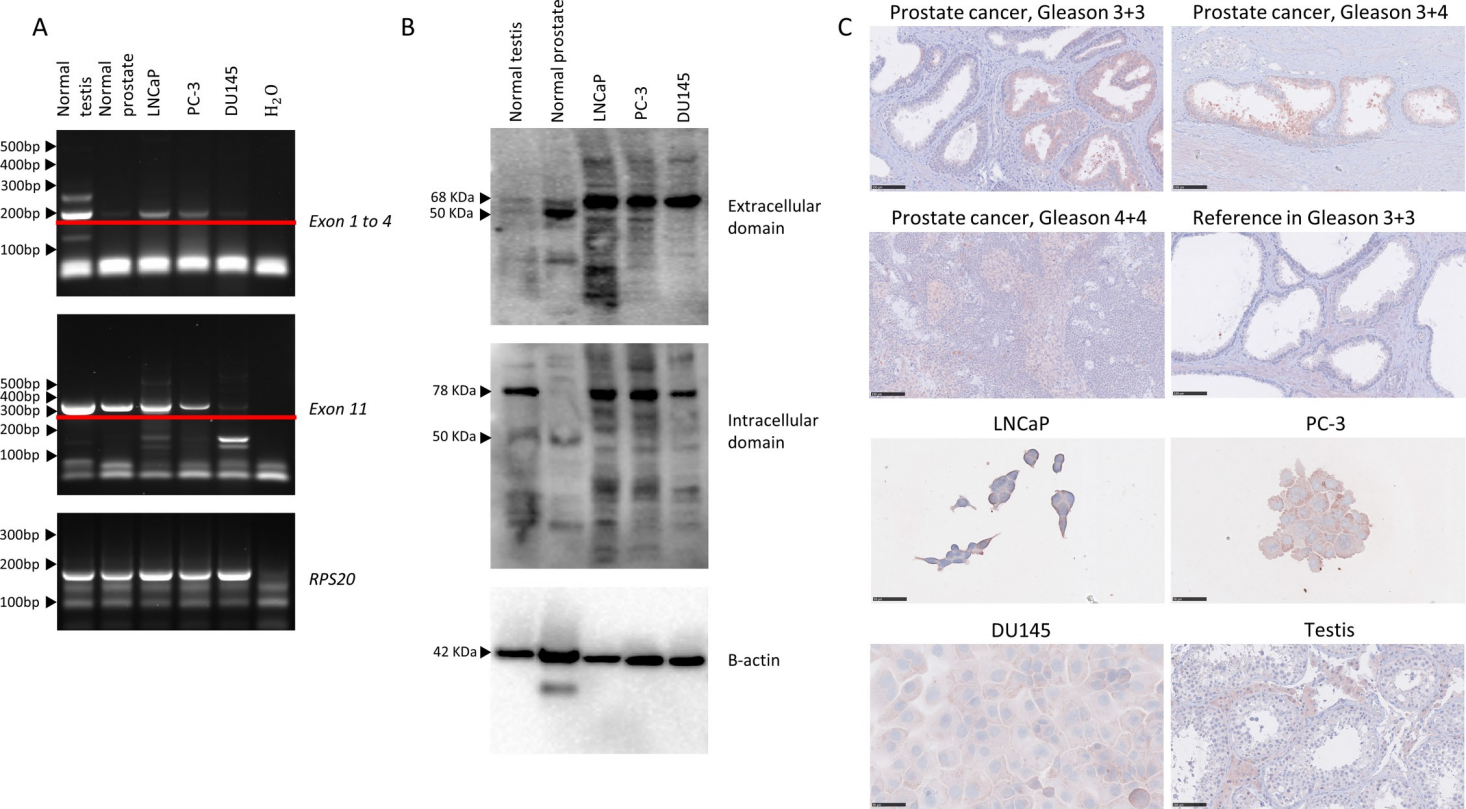
Expression of LHCGR in normal prostate, PCa and PCa cell lines. (A) RT-PCR expression of LHCGR in prostate cancer cell lines, normal testis and normal prostate. Note, a distinction is made between a primer pair targeting exon 1 to 4 and a primer pair targeting exon 11 of the *LHCGR*. Marked with the red line are the expected and sequenced bands. 5μl cDNA loaded for both *LHCGR* primer pairs and 2μl cDNA loaded for *RPS20* (B) Western blot with antibodies targeting the extracellular domain and the intra cellular domain of LHCGR. (C) IHC expression of LHCGR in PCa tissue samples and cell lines (LNCaP, PC-3 and DU145). Testis tissue sample is included as a positive control.

**Fig 2:**
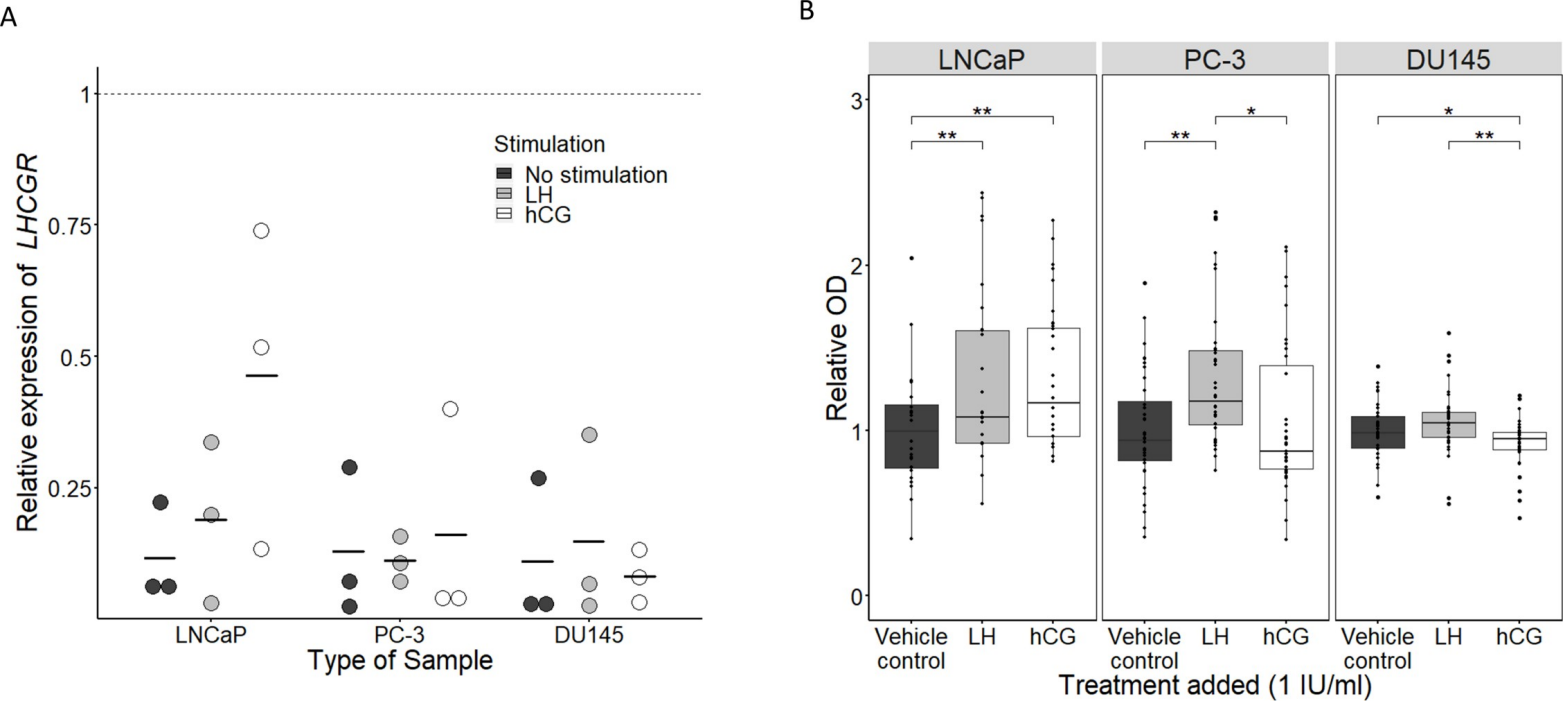
Effects of LH and hCG treatment on three prostate cancer cell lines (LNCaP, PC-3 and DU145). (A) Dot plot showing the mRNA expression (ΔΔCt) of the *LHCGR* relative to *RPS20* per cell line and type of stimulation. Expression of *LHCGR* is compared to the normal testis (dotted line). Solid line represents the mean expression of *LHCGR*. N = 3 of biological replicates, each dot consists of three technical replicates, 4μl cDNA loaded for each technical replicate. (B) Boxplot showing cell proliferation after stimulation with either LH or hCG at 1 IU/ml in the three prostate cancer cell lines. The box center represents the median, the edges of the box the inter quartile range (IQR) and the whiskers are drawn until the last containing value within 1.5*IQR, dots of individual measures plotted to show distribution N = 36 biological replicates. Significance was determined by ANOVA and post-hoc Welch two sample t-test with * P≤0.05 and ** P<0.01.

RT-PCR analysis using primers spanning exon 1–4 and 11 showed that mRNA of both extracellular and intracellular *LHCGR* was present in the LNCaP cell line (lymph node metastasis derived, hormone sensitive), in the PC-3 cell line (bone metastasis derived, hormone insensitive but AR expressing) and in the DU145 cell line (central nervous system metastasis derived, hormone insensitive) (Fig 1A). All bands marked were sequenced and validated as *LHCGR*. WB showed that three fragments of LHCGR (50, 68 and 75 kD) were expressed in testis tissues and cell lines (Fig 1B). We also tested the LHCGR antibodies on normal testis and PCa tissue specimens by IHC, which showed a specific membranous/cytoplasmic Leydig cell specific expression in the testis, while in human PCa tissue LHCGR was expressed in cytoplasm of the PCa cells and in the cytoplasm of all investigated cell lines (Fig 1C, S3 Fig). The expression of LHCGR did not seem to depend on Gleason score in human PCa tissue. LHCGR was only expressed in a small proportion of prostatic glandular epithelial cells (Fig 1C). Expression of *LHCGR* mRNA was lower in the PCa derived cell lines compared with the testis. Interestingly, LH treatment had no effect on *LHCGR* mRNA levels in any of the investigated cell lines (Fig 2A).

### LHCGR agonist alters cell proliferation in vitro

Stimulation of LNCaP and PC-3 cells with 1 IU/ml LH for 48 hours increased proliferation 1.29-fold (P = 0.007) and 1.33-fold (P = 0.0007), respectively, compared with vehicle controls. Proliferation was also increased in LNCaP cells upon stimulation with 1 IU/ml hCG for 48 hours (1.30-fold increase, P = 0.001, Fig 2B). In contrast, reduced proliferation was found after treatment with 1 IU/ml hCG for 48 hours in the DU145 cell line (0.93-fold decrease, P = 0.05, Fig 2B). When comparing treatment with similar doses of LH and hCG (for 48 hours) significantly more proliferating cells were found following LH stimulation in both PC-3 and DU145 cells (1.25-fold increase, P = 0.01 and 1.13-fold increase, P = 0.003, respectively). In a less sensitive viability assay (due to dependency on metabolic activity), no difference in the number of viable cells could be detected after LH or hCG stimulation in the PC-3 and LNCaP cell lines, but a significant decrease in viability was found after 48h of hCG and LH stimulation compared to vehicle control in the DU145 cell line (0.88-fold decrease, P<0.01, 0.93-fold decrease, P<0.01) (S1 Fig).

### Expression of steroidogenic enzymes and steroidogenesis in PCa derived cell lines

**Fig 3:**
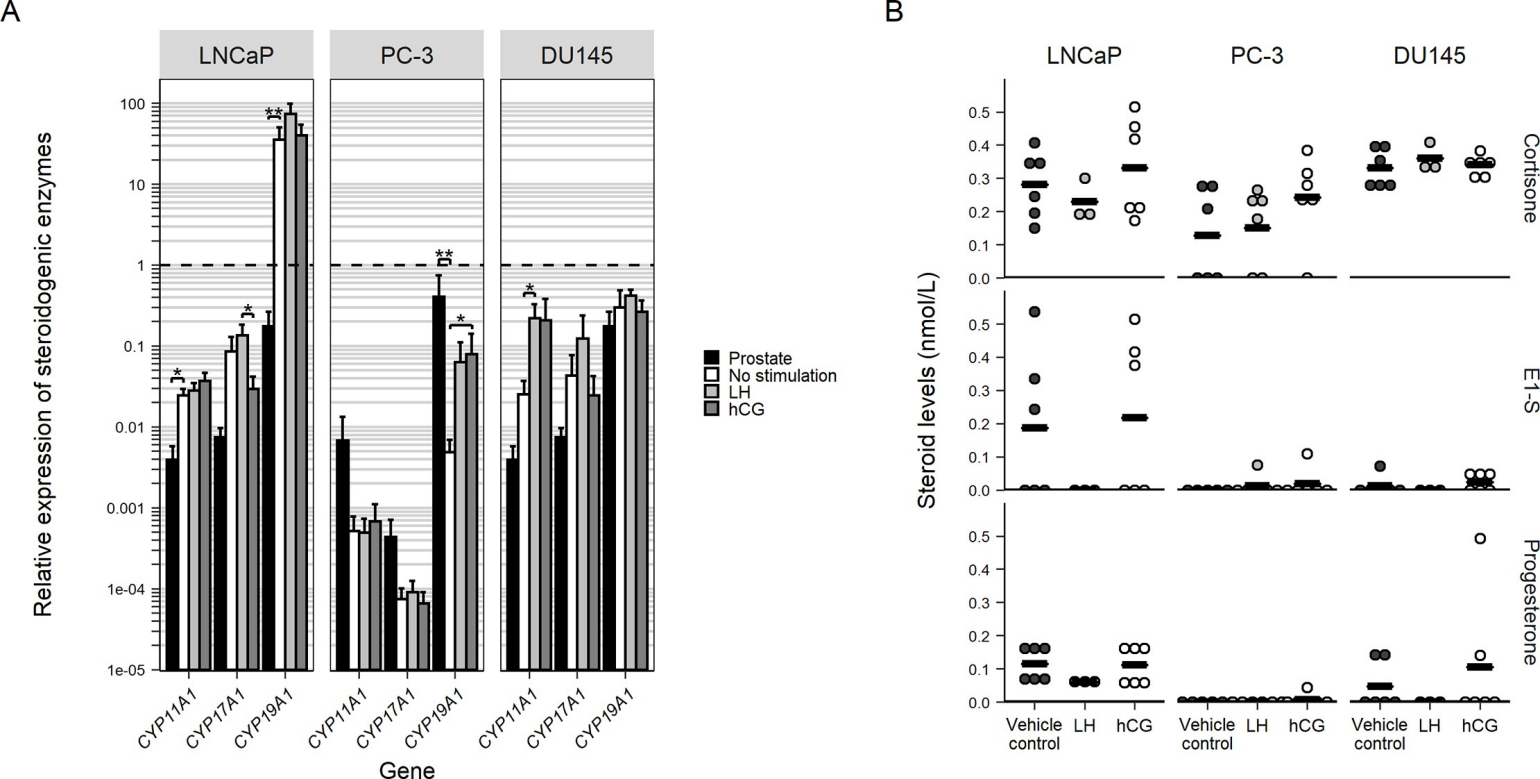
Expression of steroidogenic enzymes and production of steroids in prostate cancer cell lines. (A) Bar plot showing mRNA expression (ΔΔCt) of the steroid enzymes *CYP11A1*, *CYP17A1 and CYP19A1* relative to *RPS20* per PCa cell lines, type of stimulation and prostate compared to expression in the testis. Whiskers represent standard error of the mean. N = 6 of biological replicates of the LNCaP and DU145 cell lines, N = 9 for the PC-3 cell line and their stimulation and N = 3 for prostate measurements, each measurement is created from three technical replicates, 1μl cDNA loaded for each technical replicate except for the prostate and PCa cell lines samples for *CYP19A1*, here 4μl cDNA was loaded. (B) Dot plot of steroid measurements in medium of the cell lines after stimulation. All dots represent a single biological replicate, black line shows mean of dots shown. Significance was determined by Kruskal-Wallis Rank Sum Test and post-hoc Wilcoxon Signed Rank Tests with * P≤0.05 and ** P<0.01.

Classic steroid enzymes required for T synthesis from cholesterol were expressed in the investigated cell lines (S2 Fig). qPCR analysis showed mostly non-significant changes in the expression of key steroidogenic enzymes after treatment with hCG or LH after 48 hours in LNCaP, PC-3 and DU145 cells. Although, LH significantly increased *CYP17A1* expression when compared with hCG stimulation in the LNCaP cell line (3.6-fold higher, P = 0.03). *CYP11A1* expression was significantly higher in unstimulated LNCaP cells (5.3-fold higher, P = 0.04) compared to normal prostate, *CYP17A1* expression seemed higher in LNCaP cells compared to normal prostate, however, but upon analysis this was not statistically significant (in LNCaP 10.6-fold higher, P = 0.15). *CYP19A1* expression was significantly higher in the LNCaP cell line compared with normal prostate (199.4-fold higher, P = 0.009) and in the PC-3 cell line *CYP19A1* was significantly higher in the normal prostate compared to unstimulated cells (83.1-fold higher, P = 0.02), while stimulation with hCG increased expression compared to the unstimulated vehicle control (16.4-fold higher, P = 0.006). *CYP11A1* expression increased upon LH stimulation in the DU145 cell line (7.6-fold higher, P = 0.03) (Fig 3A).

Analysis of the media from cell cultures showed that cortisone, E1-S and progesterone were found in low amounts in control samples (Fig 3B), while T, corticosterone, DHEAS, androstenedione, 11-deoxycortisol and 17-OHP were below detection limit in the vehicle control treated samples, cortisol was only found in the PC-3 samples but was not significantly altered by stimulation. No statistically significant changes were found following 48h hCG stimulation for any of the steroids that were measurable in the control samples (Fig 3B). However, LH stimulation for 48h decreased progesterone to 53 percent of vehicle treated (P = 0.07) and resulted in levels of E1-S in media from LNCaP cells below the detection limit. In the DU145 media progesterone and E1-S became undetectable after 48h of LH stimulation (Fig 3B).

### Measurements of LHCGR in serum from PCa patients

We analyzed LHCGR in serum from 157 men with a benign prostate, localized PCa, locally advanced non-metastatic PCa, lymph-node positive PCa, de-novo metastatic PCa, or CRPC at the time of blood sample. Follow-up time for all patients was 5.9 (1.5–6.9) years (median (inter quartile range)). In all groups the serum levels of LHCGR were above level of detection in approximately two thirds of the patients. For further analysis patients were separated based on type of treatment and divided in three groups of above median measurable LHCGR, below median measurable LHCGR and undetectable LHCGR. Clinical and histopathological variables are shown in Table 2, PSA was higher in the undetectable LHCGR group compared to the measurable LHCGR groups for men treated with castration, but not significantly.

**Table 2:** Patient characteristics of groups based on initial treatment and LHCGR measurability. Showing important PCa prediction factors.

LHCGR measurements		Radical Prostatectomy as baseline treatment			Castration as baseline treatment		
		Undetectable (n = 27)	Below median (n = 20)	Above median (n = 20)	Undetectable (n = 13)	Below median (n = 10)	Above median (n = 10)
**Age (mean ± SEM)**		63.1 ±1.2	65.1 ±1.0	65.1 ±1.1	73.8 ±2.5	69.1 ±2.9	69.6 ±3,4
**PSA (ng/ml; mean ± SEM)**		10.2 ±1.8	14.6 ±2.3	10.5 ±1.7	383.3 ±222.4	182.7 ±72.2	127.6 ±43.7
**Tumour size (n, %)**	T1	8 (30)	7 (35)	9 (45)	1 (8)	0 (0)	0 (0)
	T2	16 (59)	13 (65)	10 (50)	0 (0)	1 (10)	5 (50)
	T3 + T4	3 (11)	0 (0)	1 (5)	12 (92)	9 (90)	5 (50)
	NA	0 (0)	0 (0)	0 (0)	0 (0)	0 (0)	0 (0)
**Gleason score (n, %)**	≤6	9 (33)	2 (10)	4 (20)	0 (0)	0 (0)	1 (10)
	3+4	12 (44)	9 (45)	13 (65)	1 (8)	1 (10)	1 (10)
*pGS for RP and bGS for castration	4+3	5 (19)	5 (25)	1 (5)	0	1 (10)	1 (10)
	≥8	1 (4)	4 (10)	2 (10)	10 (77)	7 (70)	7 (70)
	NA	0 (0)	0 (0)	0 (0)	2 (15)	1 (10)	0 (0)
**D’Amico score (n, %)**	Low	6 (22)	0 (0)	3 (15)	0 (0)	0 (0)	0 (0)
	Intermediate	11 (41)	10 (50)	7 (35)	0 (0)	0 (8)	0 (0)
	High	10 (37)	10 (50)	10 (50)	13 (100)	10 (100)	10 (100)
**Disease stage (n, %)**	Localized	20 (74)	11 (55)	16 (80)	0 (0)	0 (0)	0 (0)
	Locally advanced	5 (19)	5 (25)	3 (15)	0 (0)	0 (0)	0 (0)
	Lymph node positive	2 (7)	4 (20)	1 (5)	3 (38.5)	0 (0)	0 (0)
	Metastatic	0 (0)	0 (0)	0 (0)	5 (23)	6 (60)	5 (50)
	Castration resistant PCa	0 (0)	0 (0)	0 (0)	5 (38.5)	4 (40)	5 (50)

**Fig 4:**
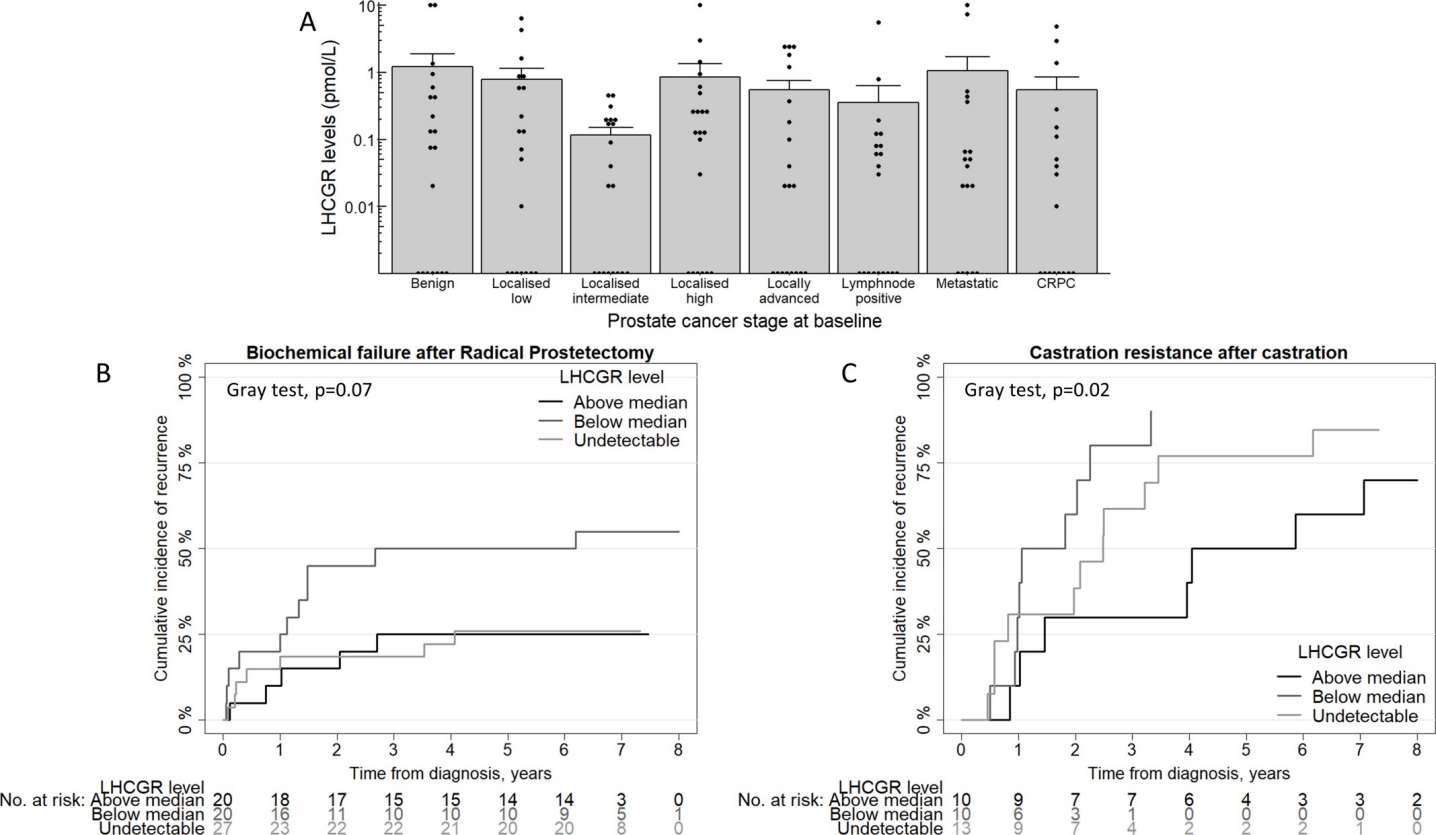
LHCGR measurements in serum. The mean + standard error of the mean of LHCGR concentration in serum is shown for different patient groups, note that it is a log10 scale (A). Localized prostate cancer disease at diagnosis is split in low-, intermediate- and high-risk groups. Each bar represents N = 20 except localized high represented with N = 21 and metastatic and castrate resistant prostate cancer (CRPC) that are represented with N = 18, that are shown as individual dots. Each dot represents a unique patient. Lower panels show cumulative incidence for recurrence in patients that underwent radical prostatectomy (B) or castration (C) as initial treatment. Lines represent the groups that are divided based on LHCGR level being above median measurable LHCGR, below median measurable LHCGR and undetectable LHCGR in serum.

Overall, no significant differences in the level of LHCGR were found between clinical PCa stages (Fig 4A). Interestingly, men with intermediate-risk localized PCa had a lower, albeit non-significant, level of LHCGR compared to both low- (85% decrease, P = 0.08) and high-risk localized (86% decrease, P = 0.14) PCa patients (Fig 4A). With univariate analysis (UVA) and multivariate analysis (MVA) we found that low levels compared to high levels of LHCGR at diagnosis were associated with a higher risk of BF after RP (UVA: HR = 2.72; 95%CI = 0.94–7.83; P = 0.06, MVA: HR = 3.05; 95%CI = 0.97–9.63; P = 0.06) and for a higher risk of castration (UVA: HR = 5.04; 95%CI = 1.65–15.39; P = 0.005, MVA: HR = 6.92; 95%CI = 1.85–25.84; P = 0.004). Furthermore, no significant difference was found for men with high-levels of LHCGR compared with men that have undetectable LHCGR on BF risk after RP (UVA: HR = 1.05; 95%CI = 0.33–3.31; P = 0.93, MVA: HR = 1.32; 95%CI = 0.35–4.89; P = 0.68) and risk of castration resistance (UVA: HR = 2.29; 95%CI = 0.87–6.06; P = 0.09, MVA: HR = 1.38; 95%CI = 0.34–5.57; P = 0.65). Other treatments such as an AR blocker or radiotherapy as baseline treatment were not assessed due to limited number of patients.

## Discussion

In this study we show that LHCGR is expressed in a fraction of the normal prostate, PCa tissue and PCa cell lines. Interestingly, LHCGR activation induces proliferation in the LNCaP and PC-3 derived PCa cell lines, while it suppresses proliferation in the DU145 PCa cell line. Additionally, our analysis suggests that serum level of LHCGR ultimately may have some prognostic value in patients with PCa, but the low number of subjects and consequent high variability in the present study warrants larger follow up studies.

Non-classical actions of LH and hCG and the presence of LHCGR outside the gonads have previously been questioned [25]. Nonetheless, in accordance with previous studies, we find LHCGR expression in the normal prostate, PCa and PCa derived cell lines at both the transcriptional and protein level, using primers and antibodies validated extensively and compared with testicular expression [14, 15]. In the WB analysis several bands were detected in addition to the bands of expected size, which is most likely due to unspecific binding of the antibodies, a major problem for GPCRs, or the presence of multiple LHCGR isoforms due to splicing or post translational modifications of LHCGR (Fig 1B). Presence of several isoforms of LHCGR may be supported by the difference in intensity between exon 11 and exon 1–4 mRNA expression of the *LHCGR* seen in the RT-PCR, but we could not support this with qPCR analysis due to un-specific binding of the exon 1–4 primers making them unsuitable for qPCR (Figs 1A and 2A). One may argue that some of the isoforms present in PCa or derived cell lines may be non-functional since some of them do not retain the intracellular/transmembrane part. However, it has previously been shown that LHCGR splice variants potentially dimerize with other LHCGR splice variants. Moreover, the follicle-stimulating hormone (FSH) receptor and the LHCGR are co-expressed in PCa cells [26–29]. In theory FSH can mediate intracellular signaling capacity together with LH and hCG indicating an unknown mechanism involving these closely related receptors. The functionality of the LHCGR in PCa derived cell lines was supported by showing the growth stimulatory effect of hCG and LH in the androgen sensitive LNCaP cells. Caution needs to be taken as factors potentially influencing growth may be present in the culture media, i.e. FBS was not removed in order to maintain normal cell growth. We hypothesized that the observed stimulatory effect of LH and hCG depends on AR expression, but no AR protein expression was detected in the PC-3 and the DU145 cells. Thus, the AR pathway cannot alone explain the differences found between these cell lines. It therefore seems unlikely that the LHCGR pathway induces the intratumoral steroidogenesis seen in late stage PCa. In here we have not been able to elucidate the mechanism through which the proliferation is altered in these advanced PCa cell lines. Future studies are needed to determine whether AR dependence in the LNCaP cell line are important by AR blocking with antagonists, such as Enzalutamide, Apalutamide or Bicalutamide and/or siRNA silencing of the AR. The observed changes in cell proliferation is in accordance with the previous finding that a lytic peptide on LH was able to decrease cell proliferation indicating binding at the PCa cell level, but functionality should be assessed by for example a cAMP assay [30]. Interestingly, LHCGR expression was found in PCa tissue, which indicates a role in PCa patients although the clinical relevance is not clear. Notably, LHCGR expression was only detected in a fraction of the prostatic cancer epithelial ducts.

PCa biology is heterogeneous with metastatic sites genetically differing from the primary tumors and multiple mechanisms driving tumor progression. However, despite being castration-resistant, androgens and AR play a key-role in the progression. Upregulation of intra-tumoral T production via upregulation of the CYP17A1 system is among the proposed mechanisms of CRPC [31, 32]. Additionally, CYP17A1 dependency is shown by the efficacy of Abiraterone acetate, that, by specifically inhibiting the CYP17A1 system, prolongs survival in men with newly diagnosed hormone-sensitive metastatic PCa and in men with metastatic CRPC [32–34]. Despite this, we did not find altered expression of key steroidogenic enzymes, including *CYP17A1* in LNCaP following LH or hCG stimulation. This is in contrast to a previous study where *CYP17A1* and *CYP11A1* was upregulated following LH stimulation in LNCaP cells [14]. The study by Pinski and colleagues also found a two-fold increased T production. In our short term 2-day culture we did not observe changes in steroidogenesis, but the cells were stimulated for ten days in the previous study, which could explain the difference. In the present study we opted for shorter treatment times because LHCGR is a GPCR, and the effects are expected to be rapid. Previous work has also shown that LHCGR may regulate cholesterol uptake via STaR in the testis, which could be of interest given the fact that statin use in men with advanced PCa have been speculated to reduce risk of PCa-related death in pharmacoepidemiologic studies [14, 35, 36]. Moreover, we found that *CYP11A1* mRNA was expressed at higher levels in both LNCaP and DU145 cell lines compared to the normal prostate sample, suggesting that there is an increased steroidogenic activity in the cell lines and thus possibly in PCa. Additionally, high expression of the aromatase (*CYP19A1*) was found in the LNCaP cell line compared to normal prostate indicating a potentially different role of the LHCGR in lymph node metastasis. We supported our findings by LC-MS/MS measurements of androgens and corticosteroids released into the media, and no LH mediated increases were found, but the product of the aromatase enzymatic activity, E1-S, was found in the media of LNCaP cells. E1-S decreased after LH stimulation in the LNCaP cell line indicating that either the E1-S is converted into active estrogens to activate the estrogen receptor or degraded by other cell mechanisms.

Interestingly, hCG has a five times potency compared LH in inducing intracellular cAMP response as well as a delayed intracellular cAMP response mediated through LHCGR in transfected cells when compared to LH [37]. Here, hCG stimulation also increases proliferation of LNCaP but, despite a higher molarity, not to the same extent as LH (1.18- vs 1.33-fold), which may be due to different intracellular signaling [37]. We also found a 1.33-fold increase in PC-3 proliferation following LH stimulation, while no change in proliferation was detected after hCG treatment. Again, indicating a difference in binding affinity or mechanism of action for hCG compared to LH. The suppressive effect of hCG but not LH on proliferation in the DU145 cell line could indicate that the growth stimulatory effect of LH is AR dependent. Although, this effect could also be mediated through unknown, T independent, direct effects on proliferation involving other signaling pathways.

Previously, it has been shown that the *LHR312 GG* genotype has a HR of 2.25 (95%C.I. 1.19–4.26; P = 0.01) compared to the *AA* genotype for PCa specific survival, which indicates that increased activity of the receptor may lead to decreased survival [13]. In this study we investigated the prognostic value of LHCGR in serum on PCa relevant outcomes using a validated ELISA platform [9, 11, 12]. Serum LHCGR levels had no apparent association with disease stage. However, it was shown that low but above detection limit baseline serum LHCGR levels was associated with BF after RP and time to castration resistance after castration-based therapy initiation in a cross-sectional study. Intriguingly we found that unmeasurable serum LHCGR is associated with decreased risk of recurrence after RP but with an increased risk of developing castration resistance after castration therapy. These results clearly need validation in larger cohorts and the true value of LHCGR in blood must be elucidated with a more sensitive detection method. Until then, caution needs to be taken interpreting the data. A U-shaped risk profile with higher or lower risks on both low and high values of a marker being similarly predictive for outcome compared to the equilibrium value are not uncommon in biology. It can be speculated that with low serum LHCGR present the LHCGR dependent steroidogenesis can still be activated/increased in contrast to high serum LHCGR where this system is already the driving force behind steroidogenesis. Ideally, future studies examining predictive value of the LHCGR should have increased sample size and combine information about LHCGR expression in prostatic tissue with serum levels of LHCGR to determine whether it is a marker of LHCGR abundance in the tumor or a proxy for something else.

In conclusion, we have confirmed that the LHCGR is present and functional in PCa. Furthermore, we show here that serum LHCGR level may be predictive for determining risk of recurrence in PCa, which needs verification in larger cohorts of PCa patients.

## Supporting information

S1 Fig Boxplot showing viability after stimulation with either LH or hCG at no stimulation (0), 0.01, 0.1 and 1 IU/ml in prostate cancer cell lines. The box center represents the median, the edges of the box the inter quartile range (IQR) and the whiskers are drawn until the last containing value within 1.5*IQR, dots of individual measures plotted to show distribution N = 48 all categories.
(TIF)

S2 Fig A RT-PCR of the steroidogenic enzymes in prostate cancer cell lines, normal testis and normal prostate. All bands are underlined at the expected size and bands were successfully sequenced for validation. 1μl cDNA was loaded for *HSD3B1*,*2*, *AKR1C3* and *RPS20*, 2μl cDNA was loaded for *CYP11A1*, *STaR*, *CYP17A1*, *CYP21A2* and *CYP11B1*,*2* and 5μl cDNA was loaded for *CYP19A1*.
(TIF)

S3 Fig Immunohistochemistry staining of the LHCGR protein at 1:7500 dilution in the testis, a prostate cancer Gleason score 3+3 and a prostate cancer Gleason score 5+4 slide with magnifications. Scale bars are 100μm for the top panel and 50μm for the bottom panel.
(TIF)

S4 Fig Western Blot of the AR (AR 441, Thermo Fisher) and B-actin in prostate cancer cell lines LNCaP, PC-3 and DU145. 15μg of protein loaded in each lane. Red boxes encircle expected size of bands.
(TIF)

S5 Fig Immunohistochemistry staining of the LHCGR protein at 1:7500 dilution in the prostate with A) positive staining and B) negative staining. Scale bars are 100μm.
(TIF)

S1 Data (XLSX)

## Abbreviations

RT-PCR: Reverse Transcription-Polymerase Chain Reaction
ANOVA: analysis of variance
IHC: immunohistochemistry
qPCR: quantitative RT-PCR
WB: western blotting
GPCR: G-protein coupled receptor
LHCGR: luteinizing hormone/choriogonadotropin receptor
hCG: human choriogonadotropin
LH: luteinizing hormone
AR: androgen receptor
CRPC: castrate-resistant prostate cancer
T: testosterone
PCa: prostate cancer
17- OHP: 17-hydroxy progesterone
DHEAS: dehydroepiandrosterone sulfate
E1-S: estrone sulphate
BF: biochemical failure
RP: radical prostatectomy
HR: hazard ratio
CI: 95% confidence intervals
UVA: univariate analysis
MVA: multivariate analysis
FSH: follicle-stimulating hormone
