# Supplementary figures and images for: Novel functions of the luteinizing hormone/chorionic gonadotropin receptor in prostate cancer cells and patients
Source: PLoS One. 2020 Sep 3;15(9):e0238814. doi: 10.1371/journal.pone.0238814 (PMC7470326; doi:10.1371/journal.pone.0238814)

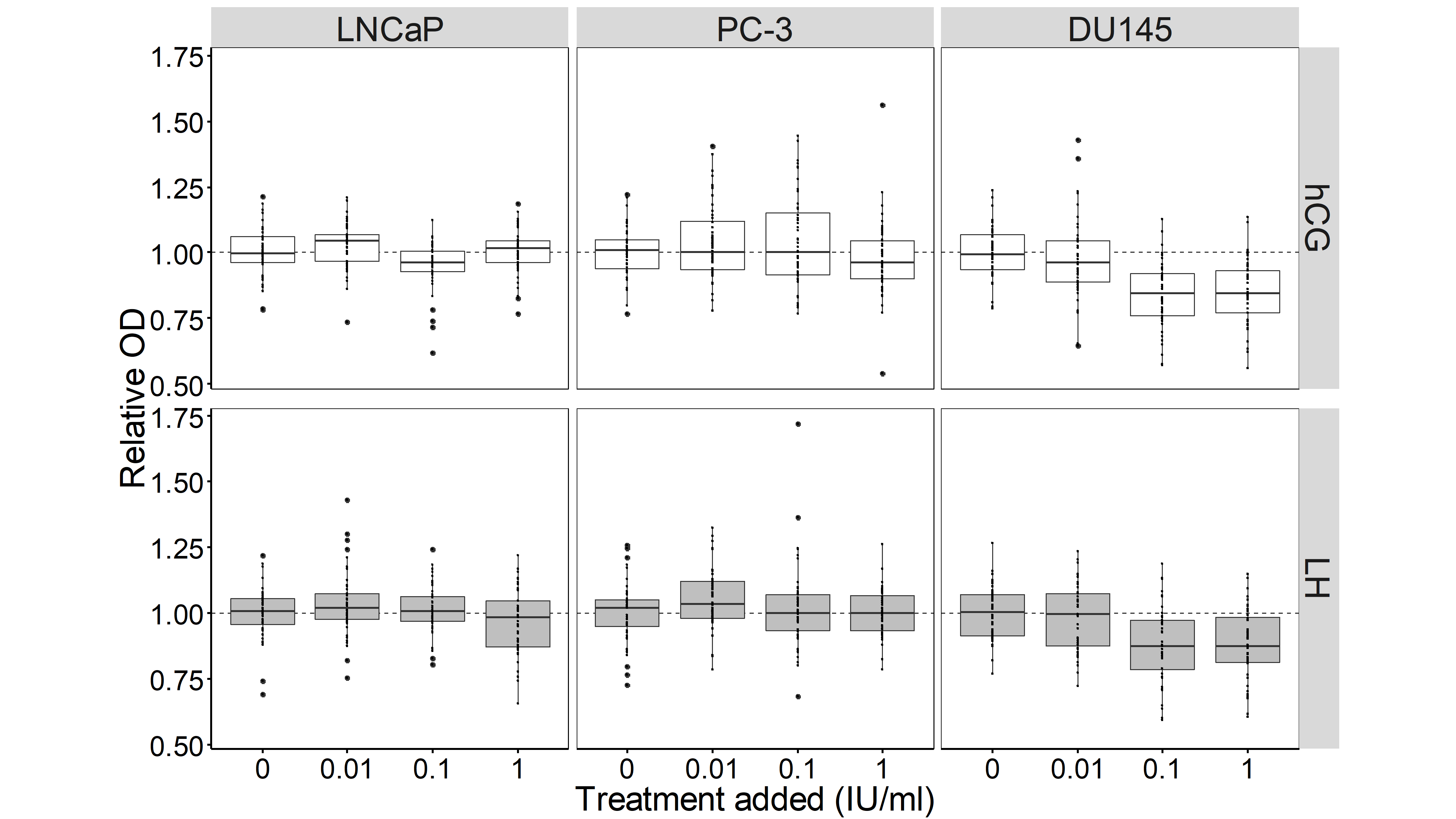

Supplement: S1 Fig — The box center represents the median, the edges of the box the inter quartile range (IQR) and the whiskers are drawn until the last containing value within 1.5*IQR, dots of individual measures plotted to show distribution N = 48 all categories. (TIF) [file pone.0238814.s001.tif]

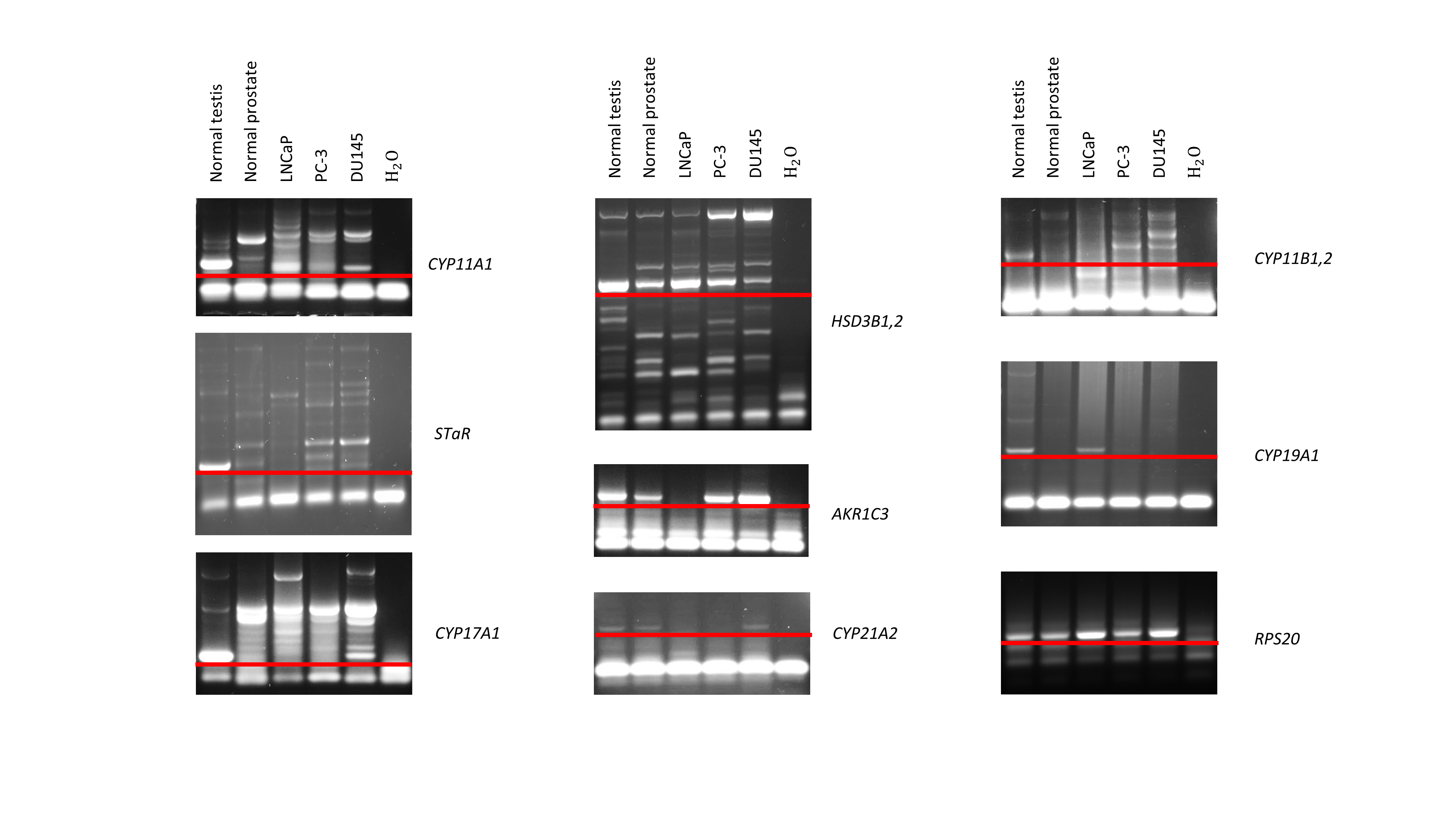

Supplement: S2 Fig — All bands are underlined at the expected size and bands were successfully sequenced for validation. 1μl cDNA was loaded for HSD3B1,2, AKR1C3 and RPS20, 2μl cDNA was loaded for CYP11A1, STaR, CYP17A1, CYP21A2 and CYP11B1,2 and 5μl cDNA was loaded for CYP19A1. (TIF) [file pone.0238814.s002.tif]

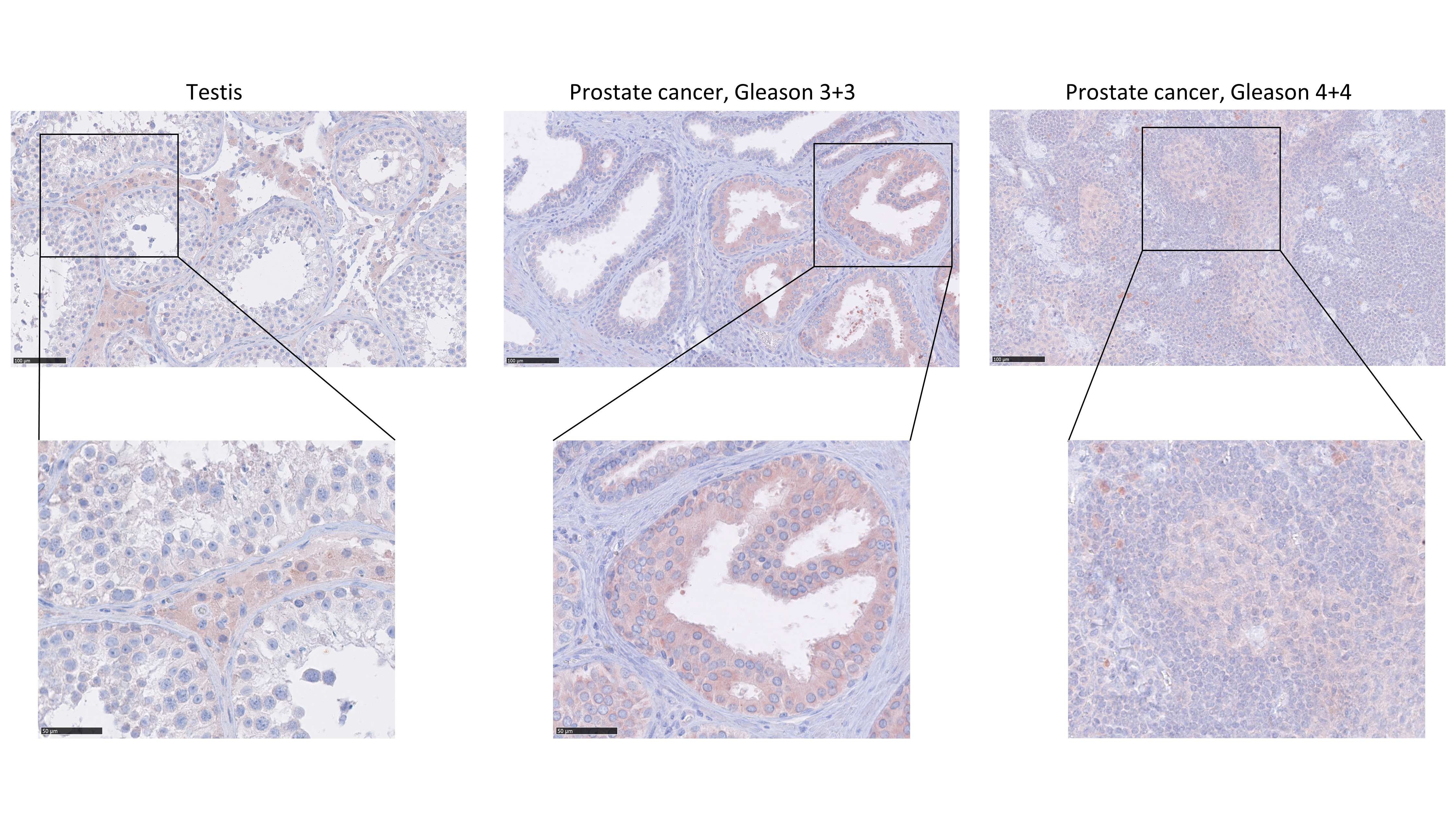

Supplement: S3 Fig — Scale bars are 100μm for the top panel and 50μm for the bottom panel. (TIF) [file pone.0238814.s003.tif]

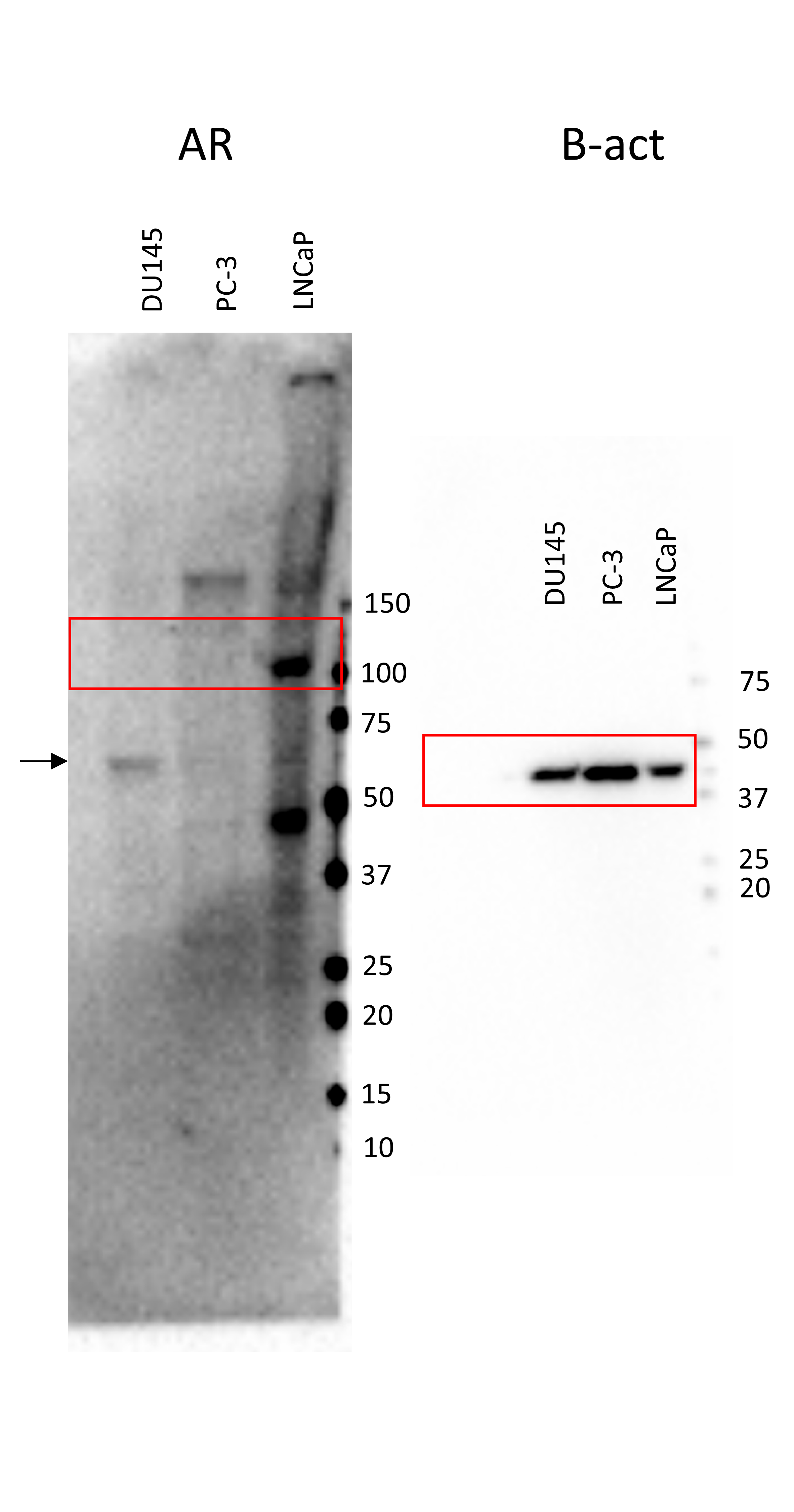

Supplement: S4 Fig — 15μg of protein loaded in each lane. Red boxes encircle expected size of bands. (TIF) [file pone.0238814.s004.tif]

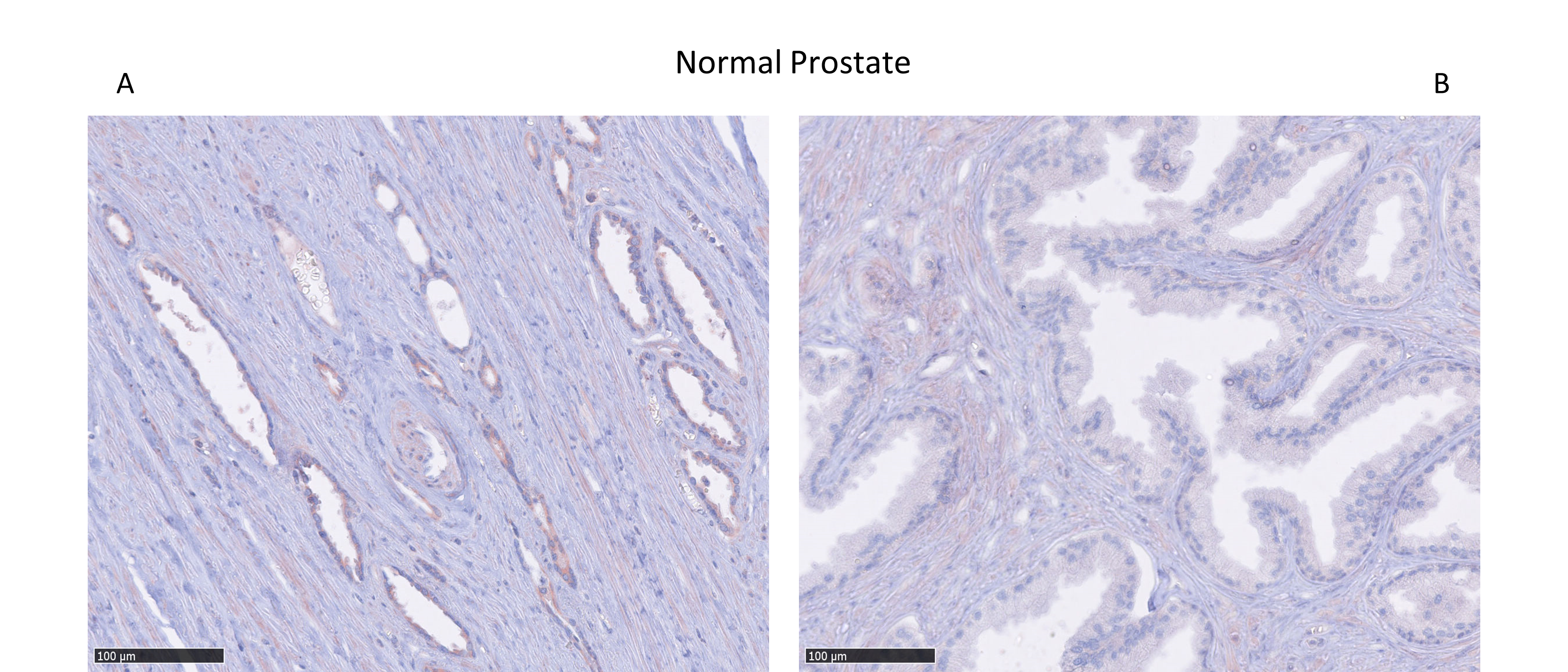

Supplement: S5 Fig — Immunohistochemistry staining of the LHCGR protein at 1:7500 dilution in the prostate with A) positive staining and B) negative staining. Scale bars are 100μm. (TIF) [file pone.0238814.s005.tif]
